# Green Hydroselenation of Aryl Alkynes: Divinyl Selenides as a Precursor of Resveratrol

**DOI:** 10.3390/molecules22020327

**Published:** 2017-02-20

**Authors:** Gelson Perin, Angelita M. Barcellos, Eduardo Q. Luz, Elton L. Borges, Raquel G. Jacob, Eder J. Lenardão, Luca Sancineto, Claudio Santi

**Affiliations:** 1 Laboratório de Síntese Orgânica Limpa-LASOL-CCQFA, Universidade Federal de Pelotas-UFPel, P.O. Box 354, 96010-900 Pelotas, RS, Brazil; angelita.barcellos@gmail.com (A.M.B.); eduardoquadros91@gmail.com (E.Q.L.); chemistry_borges@yahoo.com.br (E.L.B.); raquel.jacob@ufpel.edu.br (R.G.J.); lenardao@ufpel.edu.br (E.J.L.); 2 Department of Pharmaceutical Sciences, Group of Catalysis and Organic Green Chemistry, University of Perugia, Via del Liceo 1, 06100 Perugia, Italy; sancineto.luca@gmail.com

**Keywords:** selenium, PEG-400, tellurium, resveratrol, chalcogen alkenes

## Abstract

A simple and efficient protocol to prepare divinyl selenides has been developed by the regio- and stereoselective addition of sodium selenide species to aryl alkynes. The nucleophilic species was generates in situ*,* from the reaction of elemental selenium with NaBH_4_, utilizing PEG-400 as the solvent. Several divinyl selenides were obtained in moderate to excellent yields with selectivity for the (*Z*,*Z*)-isomer by a one-step procedure that was carried out at 60 °C in short reaction times. The methodology was extended to tellurium, giving the desired divinyl tellurides in good yields. Furthermore, the Fe-catalyzed cross-coupling reaction of bis(3,5-dimethoxystyryl) selenide **3f** with (4-methoxyphenyl)magnesium bromide **5** afforded resveratrol trimethyl ether **6** in 57% yield.

## 1. Introduction

Chalcogen alkenes are attractive key intermediates in the synthesis of important compounds. They are useful synthons to access conjugated or isolated olefins with total control in the geometry of the double bond [1,2,3,4,5]. For instance, the iron- or nickel-catalyzed cross-coupling of divinyl chalcogenides with Grignard reagents [6], the direct coupling with terminal alkynes in the presence of a nickel/CuI catalyst [7], or similar transformations [8] are useful procedures to access functionalized alkenes. The synthesis of novel selenium heterocycles based on the reaction of selenium dichloride with divinyl sulfide [9,10,11] or divinyl selenide [12] was also reported. 

**Scheme 1:**
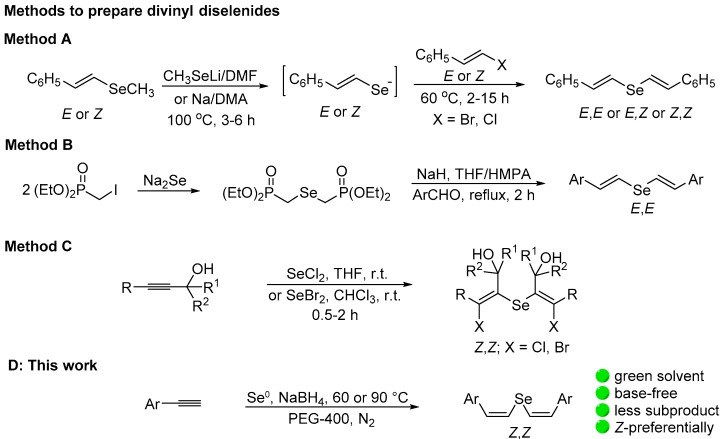
Previous general methods to prepare divinyl selenides and the present work.

Divinyl selenides however, are less studied compared to synthetic intermediates, probably due to the limited number of general methods to prepare them in a selective way. They can be prepared by a stereoconservative three-step reaction of in situ generated vinyl selenide anions with (*E*)- or (*Z*)-bromo styrenes (Scheme 1A) [13]. Divinyl selenides of (*E*,*E*)-configuration were predominantly formed by the Wittig–Horner reaction of chalcogen diphosphonate derivatives with aldehydes [14,15,16] (Scheme 1B). Both approaches are very robust, despite the low atom efficiency and a high *E* factor [17]. A more atom-economic approach of preparing divinyl selenides and tellurides involves the electrophilic addition of chalcogen halides, such as selenium dichloride or dibromide and tellurium tetrachloride, to alkynes (Scheme 1C). In these reactions, however, halo-substituted products are obtained and a reduction step is necessary to prepare the halogen-free divinyl chalcogenides [18,19,20,21,22]. *Z*,*Z*-bis(acylvinyl) selenides were exclusively obtained in moderate yields by the reaction of sodium selenide with ethynyl ketones derived from aromatic ketones. Only four examples were prepared and when bromoethynyl ketones were used, 1,3-diselenetanes were obtained instead of the respective divinyl selenides [23].

(*Z*,*Z*)-Divinyl tellurides were regio- and stereoselectively prepared by the hydrochalcogenation of alkynes via the *anti*-addition of Na_2_Te, generated in situ from Te^0^/NaBH_4_/EtOH in the presence of aqueous NaOH [24,25,26]. Alternatively, K_2_Te was used as nucleophile in the reaction with acetylene. The telluride anion was generated by heating a mixture of elemental tellurium and KOH in HMPA/H_2_O as the solvent at 110–120 °C under pressure [27]. These methods, however, are restricted to divinyl tellurides and require heating for a long time in the presence of a strong base (refluxing for up to 48 h), which is a problem with sensible substrates.

In recent years, new synthetic methodologies have been developed aiming to increase the efficiency and yields under mild reaction conditions [28,29]. In this regard, polyethylene glycol (PEG) has been extensively used as a thermally stable, nontoxic, cheap, and non-volatile solvent in the synthesis of organochalcogen compounds [30]. Some of us have recently described the in situ generation of chalcogenolate anions using the system RYYR/NaBH_4_/PEG-400 (Y = S, Se, Te) and their use in selective Michael additions, the hydrochalcogenation of alkynes [31,32,33], and the ring-opening of non-activated aziridines [34].

On the other hand, resveratrol (3,4′,5-trihydroxystilbene) is a stilbene-type polyphenolic product that is present in grapes, peanuts, mulberries, and particularly in red wine [35]. Resveratrol and methoxylated analogues are well known due to their multiple biological activities, including powerful antioxidant properties, which are associated with anti-inflammatory [36], anticancer [37], antibacterial [38] and neuroprotective effects [39]. However, these compounds are obtained only in small amounts by the extraction of plants; therefore, the development of general and simple synthetic strategies for the preparation of stilbenoids has become an important task [40,41,42,43].

As mentioned above, despite the high versatility of divinyl selenides in organic synthesis, the methods of preparing them in a selective way are limited and atom-intensive. Thus, the need for efficient and selective methods to access this class of compounds is imperative. Aiming to contribute to fill this gap, we present here our full results on the hydroselenation of aryl alkynes using the system Se^0^/NaBH_4_/PEG-400 to prepare (*Z*,*Z*)-divinyl selenides (Scheme 1D). The possibility of exploring the use of divinyl selenide in the synthesis of resveratrol trimethyl ether was also investigated.

## 2. Results and Discussion

In our preliminary experiments, we chose phenylacetylene **1a** and selenium powder **2a** as standard starting materials to perform the optimization studies under a N_2_ atmosphere and using PEG-400 as the solvent (Table 1). We evaluated the influence of temperature and the reaction time. Firstly, a mixture of selenium **2a** (0.5 mmol) and NaBH_4_ (0.8 mmol) in PEG-400 (3.0 mL) was stirred at room temperature to generate in situ the nucleophilic selenium species. The selenium reduction was accompanied by a change in the color of the reaction mixture, from grey to colorless (30 min of stirring). After this time, phenylacetylene **1a** (1.0 mmol) was added in the reaction vessel and the desired product **3a** was obtained in 12% yield after 12 h at room temperature (Table 1, Entry 1). By increasing the temperature to 40 °C, the reaction proceeds smoothly and the desired product **3a** was obtained in 42% yield in 12 h (Table 1, Entry 2). The temperature had a positive influence in the reaction of the sodium selenide with phenyl acetylene, and a similar 40% yield of **3a** was obtained in 2 h at 60 °C (Table 1, Entry 3). Next, we investigated the reaction time and observed that 5 h gave the best result, with the desired divinyl selenide **3a** being isolated as a mixture of (*Z*,*Z*)- and (*Z*,*E*)-isomers (*Z*,*Z*:*Z*,*E* ratio of 80:20) in 92% yield (Table 1, Entries 4 and 5). An additional test was performed, setting a time of 5 h and increasing the temperature to 80 °C; however, the yield was lower than that under milder conditions (Table 1, Entry 6). Thus, analyzing the results showed in Table 1, we established the best reaction conditions as being the previous reaction of selenium **2a** (0.5 mmol) and NaBH_4_ (0.8 mmol) in PEG-400 (3.0 mL) at 60 °C under N_2_ for 30 min. Afterward, phenylacetylene **1a** (1.0 mmol) is added dropwise and the resulting mixture is stirred for additional 5 h at the same temperature. 

In order to demonstrate the efficiency of this protocol, we attempted to apply the designed method to other aryl alkynes and the results are depicted in Table 2. A closer inspection of Table 2 reveals that our protocol worked well for a range of substrates, delivering products **3a**–**f** in good to excellent yields and high selectivity to the (*Z*,*Z*)-isomer. The reactions are sensitive to the electronic effect of the aromatic ring in the alkynes. The presence of strong electron-releasing group CH_3_O- in acetylenes **1c** and **1f** reduced the reactivity and a higher temperature (90 °C) was necessary to give good yields of the respective divinyl selenides **3c**,**f** (Table 2, Entries 3 and 6). This deactivating effect was less pronounced in 4*-*tolyl acetylene **1b**, which reacted under the standard conditions to afford the desired divinyl selenide **3b** after 5 h in 82% yield. In this example, a small amount of the (*E*,*E*)-isomer was detected in addition to (*Z*,*Z*)- and (*Z*,*E*)-ones (Table 2, Entry 2). The reaction is less affected by the presence of electron-withdrawing groups. 3,4-Dichlorophenyl acetylene **1e** afforded the respective (*Z*,*Z*)-divinyl selenide **3e** in 93% yield as the only isomer (Table 2, Entry 5), while 4-ethynylbenzonitrile **1d** produced **3d** in 74% yield as a mixture of (*Z*,*Z*)-, (*Z*,*E*)- and (*E*,*E*)-isomers in a 53:43:4 ratio (Table 2, Entry 4). In additional studies, the performance of the reaction using 1,1-dimethylpropargyl alcohol **1g** and hept-1-yne **1h** was studied; however, there was no formation of the expected products and the starting materials were recovered (Table 2, Entries 7 and 8).

**Scheme 2:**
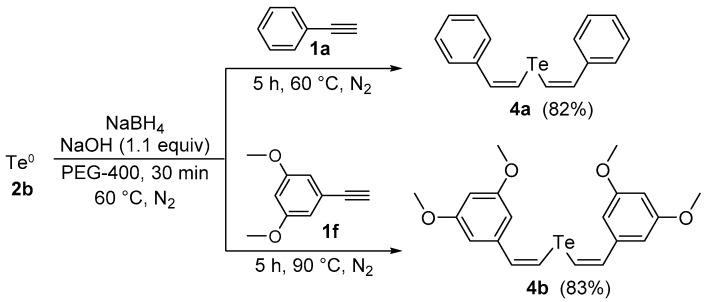
Synthesis of divinyl tellurides by hydrotelluration of aryl alkynes.

Afterward, we examined the possibility of using our protocol to prepare the divinyl tellurides analogues **4**. Firstly, we performed the reaction tellurium powder (0.5 mmol) with NaBH_4_ (0.8 mmol) in PEG-400 (3.0 mL) at 60 °C under N_2_ atmosphere until consumption of the tellurium (30 min). Then, phenylacetylene **1a** (1.0 mmol) was added and the mixture was stirred under N_2_ at the same temperature for an additional 5 h, affording the desired divinyl telluride **4a** in 40% yield (*Z*,*Z*-isomer, only). Interested in the excellent selectivity and aiming to improve the yield, the reaction was repeated in the presence of NaOH (1.1 equiv), similarly described by Tucci and co-workers in a closely related reaction [24], affording product **4a** in 82% yield after 5 h at 60 °C. By using the same strategy, bis(3,5-dimethoxystyryl) telluride **4b** was obtained in 83% yield from 3,5-dimethoxyphenylacetylene **1f** after 5 h at 90 °C (Scheme 2).

**Scheme 3:**
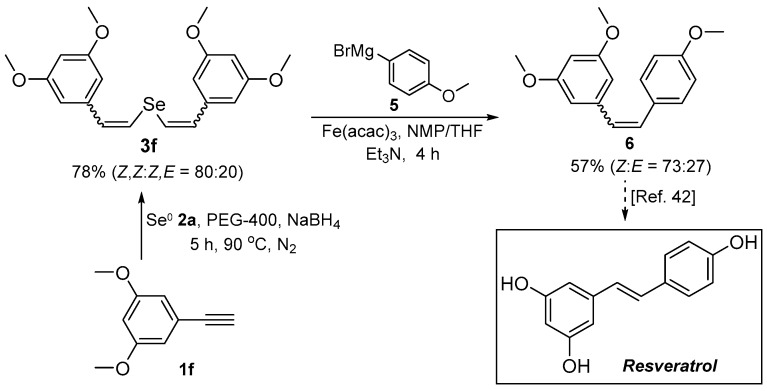
Synthesis of resveratrol trimethyl ether **6**.

In order to demonstrate the synthetic versatility of divinyl selenides, the Fe-catalyzed cross-coupling of bis(3,5-dimethoxystyryl) selenide **3f** with (4-methoxyphenyl)magnesium bromide **5** was investigated for the synthesis of resveratrol trimethyl ether **6**. Following the synthesis and purification of **3f**, it was reacted with Grignard’s reagent **5** in the presence of catalytic Fe(acac)_3_ at room temperature to afford, after 4 h, the expected resveratrol trimethyl ether **6** in 57% yield (Scheme 3). The reaction was stereoconservative, with compound **6** being obtained as a *Z*/*E* mixture (*Z*:*E* ratio of 73:27). The sequence shown in Scheme 3 is a straightforward way to polymethoxylated stilbenes, which are themselves biologically active and pharmaceutically relevant precursors (Scheme 3).

## 3. Materials and Methods

### 3.1. General Information

The reactions were monitored by thin layer chromatography (TLC), which were performed using Merck (Merck, Darmstadt, Germany) silica gel (60 F_254_), with a 0.25 mm thickness. For visualization, TLC plates were either exposed to UV light, or stained with iodine vapor or in a 5% vanillin solution in 10% aqueous H_2_SO_4_ and heat. Column chromatography was performed using Merck Silica Gel (230–400 mesh). High-resolution mass spectra (HRMS) were recorded in positive ion mode (ESI) using a Bruker microQTOF spectrometer (Bruker, Billerica, MA, USA). Low-resolution mass spectra were obtained with a Shimadzu GC-MS-QP2010 mass spectrometer (Shimadzu Corporation, Kyoto, Japan). NMR spectra were recorded with Bruker DPX (Bruker). (^1^H-NMR = 400 and 500 MHz; ^13^C-NMR = 100 and 126 MHz) instruments using CDCl_3_ as solvent and calibrated using tetramethylsilane (TMS) as internal standard. Coupling constants (*J)* were reported in Hertz and chemical shifts (δ) in ppm. The NMR spectra are found in the Supplementary Materials. The reagents (substituted alkynes, sodium tetrahydroborate, elemental chalcogen) and PEG-400 were purchased from Sigma-Aldrich (Sigma-Aldrich, St. Louis, MO, USA).

### 3.2. General Procedure for the Preparation of Divinyl Selenides ***3a**–**f***

In a two necked round bottomed flask under nitrogen atmosphere, sodium selenide was generated by reaction of elemental selenium (Se^0^, 0.5 mmol) with sodium tetrahydroborate (0.8 mmol, 0.030 g) in PEG-400 (3.0 mL) at 60 °C. After 30 min, a colorless solution was obtained and the corresponding alkyne was added (1.0 mmol). After stirring for 5 h at 60 or 90 °C, the reaction mixture was quenched with water (10.0 mL) and extracted with ethyl acetate (3 × 15.0 mL). The combined organic layers were dried over MgSO_4_, filtered and concentrated under vacuum. The residue was purified by column chromatography over silica gel eluting with hexanes to yield the products as an inseparable mixture of isomers. All compounds were properly characterized by MS, ^1^H-NMR, and ^13^C-NMR, and the isomers ratios were determined by GC and NMR.

*Bis-(Z,Z)-styryl selenide* **3a**: Yield: 0.132 g (92%) [13]; Orange solid; ^1^H-NMR (CDCl_3_, 500 MHz) δ (ppm): 7.20–7.38 (m, 10H), 6.92 (d, *J* = 10.4 Hz, 2H), 6.64 (d, *J* = 10.4 Hz, 2H). MS: *m/z* (rel. int.) 286 (M^+^, 22.2), 205 (100), 102 (36.7), 91 (42.8), 77 (50.4). *Z*,*E*-**3a**: [13] ^1^H-NMR (CDCl_3_, 500 MHz) δ (ppm): 7.14 (d, *J* = 10.4 Hz, 1H), 7.08 (d, *J* = 15.4 Hz, 1H), 6.85 (d, *J* = 15.4 Hz, 1H), 6.75 (d, *J* = 10.4 Hz, 1H), other peaks were overlapped with those of *Z*,*Z*-**3a**. MS: *m*/*z* (rel. int.) 286 (M^+^, 25.0), 205 (100), 102 (35.3), 91 (41.4), 77 (44.1). ^13^C-NMR (125 MHz, CDCl_3_) δ (ppm): (*Z*,*Z* + *Z*,*E*) 137.0, 136.7, 134.7, 134.1, 130.5, 130.2, 128.61, 128.57, 128.4, 128.2, 127.9, 127.6, 127.3, 126.3, 125.9, 123.4, 121.3, 119.5. HRMS (ESI): *m*/*z* calcd. for C_16_H_14_Se [M + H]^+^: 287.0339, found: 287.0328.

*Bis-(Z,Z)-4-methylstyryl selenide* **3b**: Yield: 0.129 g (82%); Orange solid; ^1^H-NMR (CDCl_3_, 400 MHz) *δ* (ppm): 7.10–7.50 (m, 8H), 6.91 (d, *J* = 10.4 Hz, 2H), 6.60 (d, *J* = 10.4 Hz, 2H), 2.34 (s, 6H). MS: *m*/*z* (rel. int.) 314 (M^+^, 38.8), 219 (100), 204 (25.9), 102 (20.4), 91 (32.4), 77 (6.6). *Z*,*E*-**3b**: ^1^H-NMR (CDCl_3_, 400 MHz) δ (ppm): 6.96 (d, *J* = 10.3 Hz, 1H), 6.86 (d, *J* = 15.6 Hz, 1H), 6.76 (d, *J* = 15.6 Hz, 1H), 6.69 (d, *J* = 10.3 Hz, 1H), other peaks were overlapped with those of *E*,*E* and *Z*,*Z-***3b**. MS: *m/z* (rel. int.) 314 (M^+^, 32.6), 219 (100), 204 (23.7), 102 (18.6), 91 (28.0), 77 (6.2). *E*,*E*-**3b**: [15] ^1^H-NMR (CDCl_3_, 400 MHz) δ (ppm): 7.04 (d, *J* = 15.8 Hz, 2H), 6.84 (d, *J* = 15.8 Hz, 2H), other peaks were overlapped with those of *Z*,*E* and *Z,Z-***3b**. MS: *m*/*z* (rel. int.) 314 (M^+^, 39.3), 219 (100), 204 (25.0), 102 (17.4), 91 (29.4), 77 (7.0). ^13^C-NMR (CDCl_3_, 100 MHz) δ (ppm): (*Z*,*Z* + *Z*,*E* + *E*,*E*) 138.0, 137.5, 137.13, 137.11, 134.31, 134.29, 134.25, 130.4, 130.1, 129.34, 129.3, 129.1, 128.22, 128.17, 127.2, 126.5, 126.3, 126.1, 125.91, 125.87, 122.5, 120.5, 118.2, 116.6, 21.3, 21.2. HRMS (ESI): *m*/*z* calcd. for C_18_H_18_Se [M + H]^+^: 315.0652, found: 315.0648.

*Bis-(Z,Z)-4-methoxystyryl selenide* **3c**: Yield: 0.133 g (77%); Orange solid; ^1^H-NMR (CDCl_3_, 500 MHz) δ (ppm): 6.72–7.34 (m, 10H), 6.52 (d, *J* = 9.9 Hz, 2H), 3.80 (s, 6H). MS: *m*/*z* (rel. int.) 346 (M^+^, 13.9), 266 (100), 235 (36.7), 77 (10.2). *Z*,*E*-**3c**: ^1^H-NMR (CDCl_3_, 500 MHz) δ (ppm): 6.61 (d, *J* = 10.2 Hz, 1H), 3.78 (s, 6H), other peaks were overlapped with those of Z,*Z*-**3c**. MS: *m/z* (rel. int.) 346 (M^+^, 14.8), 266 (100), 235 (35.6), 77 (10.7). ^13^C-NMR (CDCl_3_, 100 MHz) δ *(*ppm): (*Z*,*Z* + *Z*,*E*) 159.2, 158.7, 134.6, 134.2, 129.9, 129.8, 129.7, 129.63, 129.6, 127.2, 127.1, 121.0, 119.2, 116.5, 114.2, 114.0, 113.8, 113.7, 55.2. HRMS (ESI): *m/z* calcd. for C_18_H_18_O_2_Se [M + H]^+^: 347.0550, found: 347.0545.

*Bis-(Z,Z)-4-cyanostyryl selenide* **3d**: Yield: 0.124 g (74%); Orange solid; ^1^H-NMR (DMSO-*d*_6_, 400 MHz) δ (ppm): 6.68–7.03 (m, 8H), 6.47 (d, *J* = 10.5 Hz, 2H), 6.30 (d, *J* = 10.5 Hz, 2H). *Z*,*E*-**3d**: ^1^H-NMR (DMSO-*d*_6_, 400 MHz) δ (ppm): 6.57 (d, *J* = 10.5 Hz, 1H), 6.52 (d, *J* = 15.1 Hz, 1H), 6.45 (d, *J* = 15.1 Hz, 1H), 6.19 (d, *J* = 10.5 Hz, 1H), other peaks were overlapped with those of *E*,*E* and Z,*E*-**3d**. *E*,*E*-**3d**: ^1^H-NMR (DMSO-*d*_6_, 400 MHz) δ (ppm): 6.08 (d, *J* = 15.6 Hz, 2H), other peaks were overlapped with those of *Z*,*Z* and Z,*E*-**3d**. ^13^C-NMR (DMSO-*d*_6_, 100 MHz) δ (ppm): (*Z*,*Z* + *Z*,*E* + *E*,*E*) 141.1, 141.0, 140.6, 132.8, 132.4, 132.33, 132.26, 130.7, 130.5, 128.9, 128.8, 128.7, 128.4, 127.2, 127.1, 126.8, 126.42, 126.36, 125.7, 125.5, 118.5, 118.4, 110.2, 109.9, 109.6, 109.4, 109.38. MS: *m/z* (rel. int.) 336 (M^+^, 19.0), 207 (100), 191 (13.8), 127 (40.4), 102 (9.0), 77 (11.6). HRMS (ESI): *m*/*z* calcd. for C_18_H_12_N_2_Se [M]^+^: 336.0166, found: 336.0150.

*Bis-(Z,Z)-3,4-dichlorostyryl selenide* **3e**: Yield: 0.196 g (93%). Yellowish solid; m.p.: 152–155 °C. ^1^H-NMR (CDCl_3_, 400 MHz) δ (ppm): 7.43–7.45 (m, 4H), 7.21 (dd, *J* = 8.2 and 2.1 Hz, 2H), 6.86 (d, *J* = 10.4 Hz, 2H), 6.73 (d, *J* = 10.4 Hz, 2H). ^13^C-NMR (CDCl_3_, 100 MHz) δ (ppm): δ 136.8, 132.7, 131.4, 130.5, 130.1, 128.6, 127.2, 124.9. MS: *m*/*z* (rel. int.) 422 (M^+^, 6.2), 207 (83.8), 40 (100). HRMS (ESI): *m*/*z* calcd. for C_16_H_10_C_l4_Se [M + H]^+^: 422.8780, found: 422.8758.

*Bis-(Z,Z)-3,5-dimethoxystyryl selenide* **3f**: Yield: 0.158 g (78%); Yellowish solid; ^1^H-NMR (CDCl_3_, 500 MHz) δ (ppm): 6.88 (d, *J* = 10.3 Hz, 2H), 6.67 (d, *J* = 10.3 Hz, 2H), 6.54 (d, *J* = 2.3 Hz, 4H), 6.38 (t, *J* = 2.3 Hz, 2H), 3.79 (s, 12H). ^13^C-NMR (CDCl_3_, 125 MHz) δ (ppm): 160.7, 138.8, 130.2, 124.0, 106.1, 100.0, 55.3. *Z*,*E-***3f**: ^1^H-NMR (CDCl_3_, 500 MHz) δ (ppm): 7.11 (d, *J* = 15.7 Hz, 1H), 6.93 (d, *J* = 10.3 Hz, 1H), 6.79 (d, *J* = 15.7 Hz, 1H), 6.77 (d, *J* = 10.3 Hz, 1H), 6.48 (d, *J* = 2.3 Hz, 4H), 6.34–6.35 (m, 2H), 3.81 (s, 12H). MS: *m*/*z* (rel. int.) 406 (M^+^, 2.8), 325 (100), 207 (45.9), 77 (15.0). HRMS (ESI): *m*/*z* calcd. for C_20_H_22_O_4_Se [M + H]^+^: 407.0762, found: 407.0740.

### 3.3. General Procedure for the Preparation of Divinyl Tellurides ***4a**,**b***

In a two-necked round bottomed flask under nitrogen atmosphere, sodium telluride was generated by reaction of elemental tellurium (Te^0^, 0.5 mmol) with sodium tetrahydroborate (0.8 mmol, 0.030 g) and NaOH (0.55 mmol, 0.022 g) in PEG-400 (3.0 mL) at 60 °C. After 30 min, a violet solution was obtained and the corresponding alkyne was added (1.0 mmol). After stirring for 5 h at 60 or 90 °C, the reaction mixture was quenched with water (10.0 mL) and extracted with ethyl acetate (3 × 15.0 mL). The combined organic layers were dried over MgSO_4_, filtered, and concentrated under vacuum. The residue was purified by column chromatography over silica gel eluting with hexanes to yield the products. All compounds were properly characterized by MS, ^1^H-NMR, and ^13^C-NMR.

*Bis-(Z,Z)-styryl telluride* **4a**: Yield: 0.138 g (82%); White solid; m.p.: 44–46 °C (Lit. [26]: 46–47 °C). ^1^H-NMR (CDCl_3_, 400 MHz) δ (ppm): 7.43 (d, *J* = 10.6 Hz, 2H), 7.33–7.36 (m, 4H), 7.22–7.24 (m, 6H), 6.99 (d, *J* = 10.6 Hz, 2H). ^13^C-NMR (CDCl_3_, 100 MHz) δ (ppm): 138.8, 137.3, 128.4, 127.6, 127.4, 108.9. MS: *m/z* (rel. int.) 336 (M^+^, 17.2), 206 (100), 91 (71.4), 77(67.1).

*Bis-(Z,Z)-3,5-dimethoxystyryl telluride* **4b**: Yield: 0.189 g (83%); Orange solid; m.p.: 76–77 °C. ^1^H-NMR (DMSO-*d*_6_, 400 MHz) δ (ppm): 7.45 (d, *J* = 10.5 Hz, 2H), 7.23 (d, *J* = 10.5 Hz, 2H), 6.42–6.47 (m, 6H), 3.76 (s, 12H). ^13^C-NMR (DMSO-*d*_6_, 100 MHz) δ (ppm): 160.6, 140.5, 136.9, 110.1, 105.0, 99.6, 55.2. MS: *m*/*z* (rel. int.) 456 (M^+^, 13.7), 325 (100), 175 (34.0). HRMS (ESI): *m*/*z* calcd. for C_20_H_22_O_4_Te [M + H]^+^: 457.0659, found: 457.0662.

### 3.4. Preparation of 3,4’,5-Trimethoxystilbene ***6***

To a two-necked 100 mL round bottomed flask under nitrogen, the dry solvent (NMP/THF 1:3, 20.0 mL), bis(3,5-dimethoxystyryl) selenide **3f** (0.406 g, 1 mmol), Et_3_N (2.5 mL) and Fe(acac)_3_ (71 mg, 20 mol %) was added. The mixture was stirred at room temperature, and after 15 min a solution of the Grignard reagent from 1-bromo-4-methoxybenzene (**5**, 10 mmol; 10.0 mL of a 1 mol/L sol. in THF) was added dropwise. The reactions were monitored by TLC until total disappearance of the starting material **3f**. After completion of the reaction, aqueous sat. NH_4_Cl (15.0 mL) was added and the reaction mixture was extracted with ethyl acetate (3 × 20.0 mL), the organic layer was washed with water and dried over MgSO_4_. After filtration, the solvent was removed under reduced pressure and the residue was purified by column chromatography on silica gel (ethyl acetate/hexanes, 10:90).

*3,4’,5-Trimethoxystilbene* **6**: Yield: 0.155 g (57%) [42]; Whitish oil; *Z*-**6**: ^1^H-NMR (CDCl_3_, 400 MHz) *δ* (ppm): 7.11 (d, *J* = 8.7 Hz, 2H), 6.66 (d, *J* = 8.7 Hz, 2H), 6.42 (d, *J* = 12.0 Hz, 1H), 6.343 (d, *J* = 2.3 Hz, 2H), 6.337 (d, *J* = 12.0 Hz, 1H), 6.22 (t, *J* = 2.3 Hz, 1H), 3.66 (s, 3H), 3.56 (s, 6H). MS: *m/z* (rel. int.) 270 (M^+^, 100), 239 (23.6), 224 (15.6), 196 (7.9), 181 (4.9), 152 (7.8), 141 (4.7), 115 (6.0), 102 (1.2) 76 (2.9). *E*-**6**: ^1^H-NMR (CDCl_3_, 400 MHz) δ (ppm): 7.33 (d, *J* = 8.8 Hz, 2H), 6.94 (d, *J* = 16.2 Hz, 1H), 6.80 (d, *J* = 16.2 Hz, 1H), 6.78 (d, *J* = 8.8 Hz, 2H), 6.55 (d, *J* = 2.3 Hz, 2H), 6.28 (t, *J* = 2.3 Hz, 1H), 3.71 (s, 6H), 3.70 (s, 3H). MS: *m/z* (rel. int.) 270 (M^+^, 100), 239 (21.3), 224 (10.8), 196 (6.5), 181 (4.1), 152 (6.0), 141 (3.5), 115 (4.2), 102 (0.9) 76 (2.4). ^13^C-NMR (CDCl_3_, 100 MHz) δ (ppm): (*Z* + *E*) 160.9, 160.5, 159.3, 158.7, 139.6, 139.4, 130.2, 130.1, 129.8, 129.5, 128.65, 128.60, 127.7, 126.5, 114.1, 113.4, 106.5, 104.3, 99.8, 99.5, 55.22, 55.18, 55.1.

## 4. Conclusions

An efficient methodology to prepare divinyl selenides from elemental selenium and arylalkynes was developed. The nucleophilic species of selenium was generated in situ from elemental selenium and NaBH_4_/PEG-400. The reactions proceeded at a gentle heating of 60 °C for only 5 h, affording the corresponding divinyl selenides in good to excellent yields with high selectivity to the *Z*,*Z*-isomer. The same strategy was used to prepare (*Z*,*Z*)-divinyl tellurides analogues from elemental tellurium, but good results were obtained only when NaOH was present in the reaction media. Divinyl selenides with the appropriate substitution pattern was used in a Fe-catalyzed cross-coupling reaction with a Grignard reagent to afford 3,4’,5-trimethoxystilbene in moderate yield. Thus, this procedure could be used to access valuable polymethoxylated stilbenes. 

## Figures and Tables

**Table 1:**
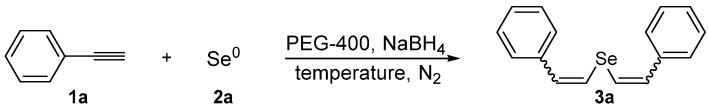
Optimization of the synthesis of divinyl selenide **3a** ^a^.

Entry	Temperature	Time (h)	Yield (%) ^b^
1	r.t.	12	12
2	40 °C	12	42
3	60 °C	2	40
4	60 °C	3	72
5	60 °C	5	92
6	80 °C	5	30

^a^ Reactions performed in the presence of **1a** (1.0 mmol), **2a** (0.5 mmol), NaBH_4_ (0.8 mmol) and solvent (3.0 mL) under N_2_ atmosphere. ^b^ Yields are given for isolated product **3a**, which was obtained as a mixture of (*Z*,*Z*)- and (*Z*,*E*)-isomers.

**Table 2:**
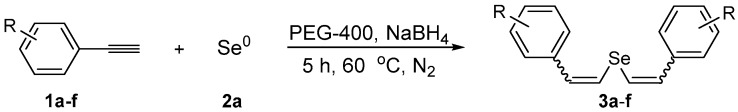
Synthesis of divinyl selenides **3** by the hydroselenation of aryl alkynes **1** ^a^.

Entry	Alkynes 1	Product 3	Yield (%) ^b^	Ratio (*Z*,*Z/Z,E/E,E*)
1	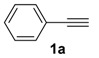	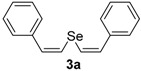	92	80/20/00 ^c^
2	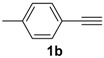	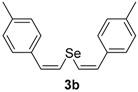	82	71/15/14 ^d^
3	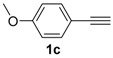	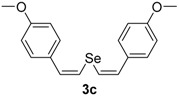	77	77/23/00 ^d,e^
4	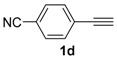	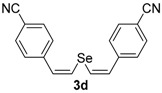	74	53/43/04 ^c^
5	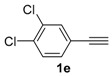	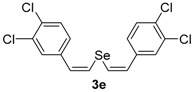	93	100/00/00 ^c^
6	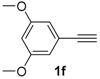	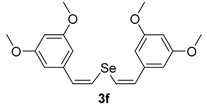	78	80/20/00 ^c,e^
7	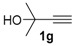	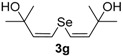	nr	-
8	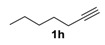	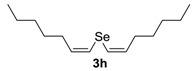	nr	-

^a^ Reactions performed in the presence of aryl alkynes **1** (1.0 mmol), selenium **2a** (0.5 mmol), NaBH_4_ (0.8 mmol), and PEG-400 (3.0 mL) under N_2_ atmosphere. ^b^ Yields are given for isolated products **3a**–**f**. ^c^ Determined by ^1^H-NMR of the purified product. ^d^ Determined by GC/MS of the purified product. ^e^ Reaction performed at 90 °C; nr = no reaction.

## Supplementary Materials

NMR spectra of synthesized compounds are available online.
