# Supplementary material for: Green Hydroselenation of Aryl Alkynes: Divinyl Selenides as a Precursor of Resveratrol
Source: Molecules. 2017 Feb 20;22(2):327. doi: 10.3390/molecules22020327 (PMC6155768; doi:10.3390/molecules22020327)
Supplement: Supplementary file 1 [file molecules-22-00327-s001.pdf]

Supporting Information: Green hydroselenation of aryl alkynes: divinyl selenides as precursor of resveratrol

Gelson Perin [1,*], Angelita M. Barcellos [1], Eduardo Q. Luz [1], Elton L. Borges [1], Raquel G. Jacob [1], Eder J. Lenardão, [1] Luca Sancineto [2] and Claudio Santi [2,*]

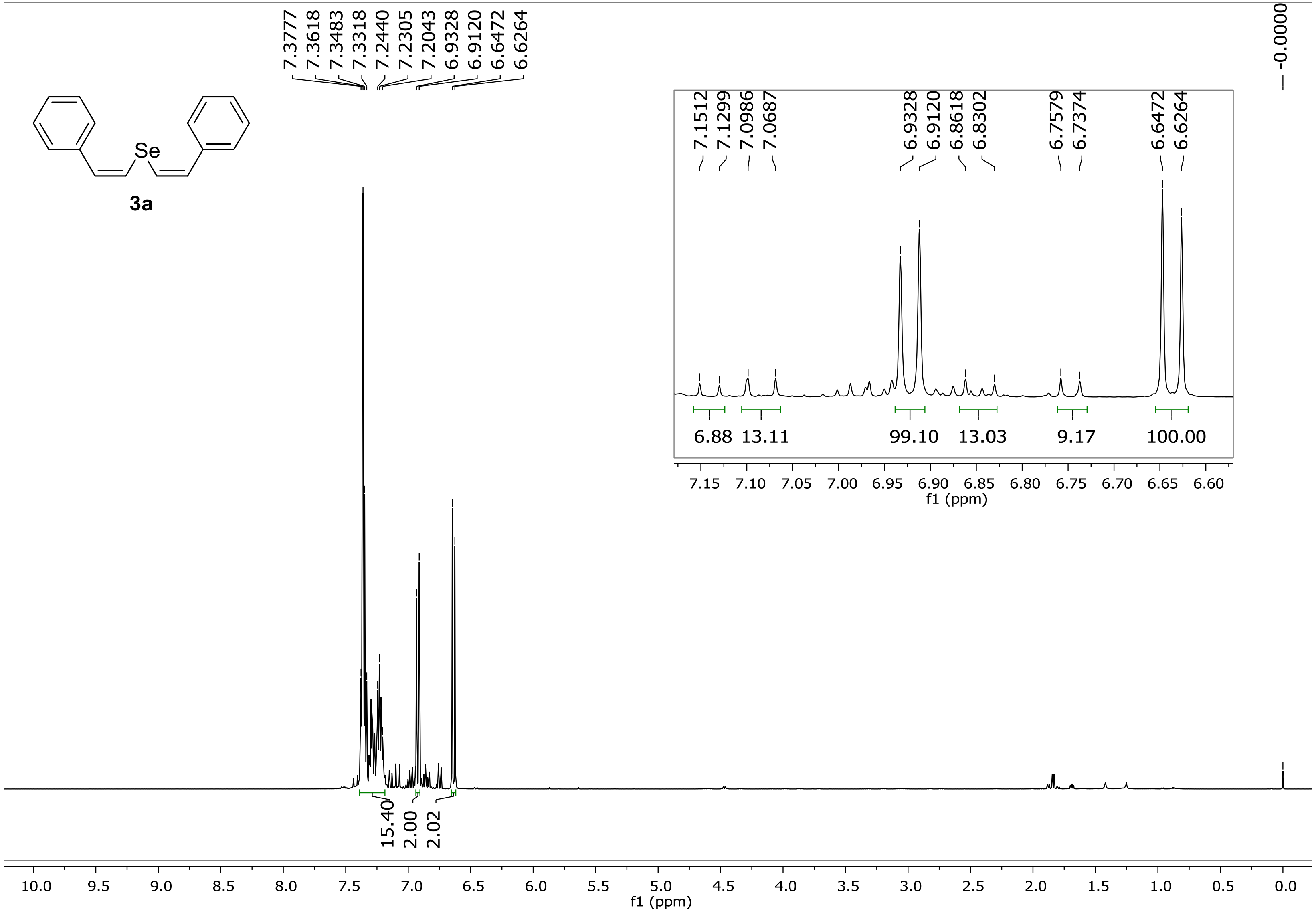

Se
3a
7.3777
7.3618
7.3483
7.3318
7.2440
7.2305
7.2043
6.9328
6.9120
6.6472
6.6264
-0.0000
7.1512
7.1299
7.0986
7.0687
6.9328
6.9120
6.8618
6.8302
6.7579
6.7374
6.6472
6.6264
6.88
13.11
99.10
13.03
9.17
100.00
7.15 7.10 7.05 7.00 6.95 6.90 6.85 6.80 6.75 6.70 6.65 6.60
f1 (ppm)
15.40
2.00
2.02
10.0 9.5 9.0 8.5 8.0 7.5 7.0 6.5 6.0 5.5 5.0 4.5 4.0 3.5 3.0 2.5 2.0 1.5 1.0 0.5 0.0
f1 (ppm)

Figure S1. ^{1}H NMR (500 MHz, $CDCl_3$) spectrum of bis-(*Z*,*Z*)-styryl selenide **3a**.

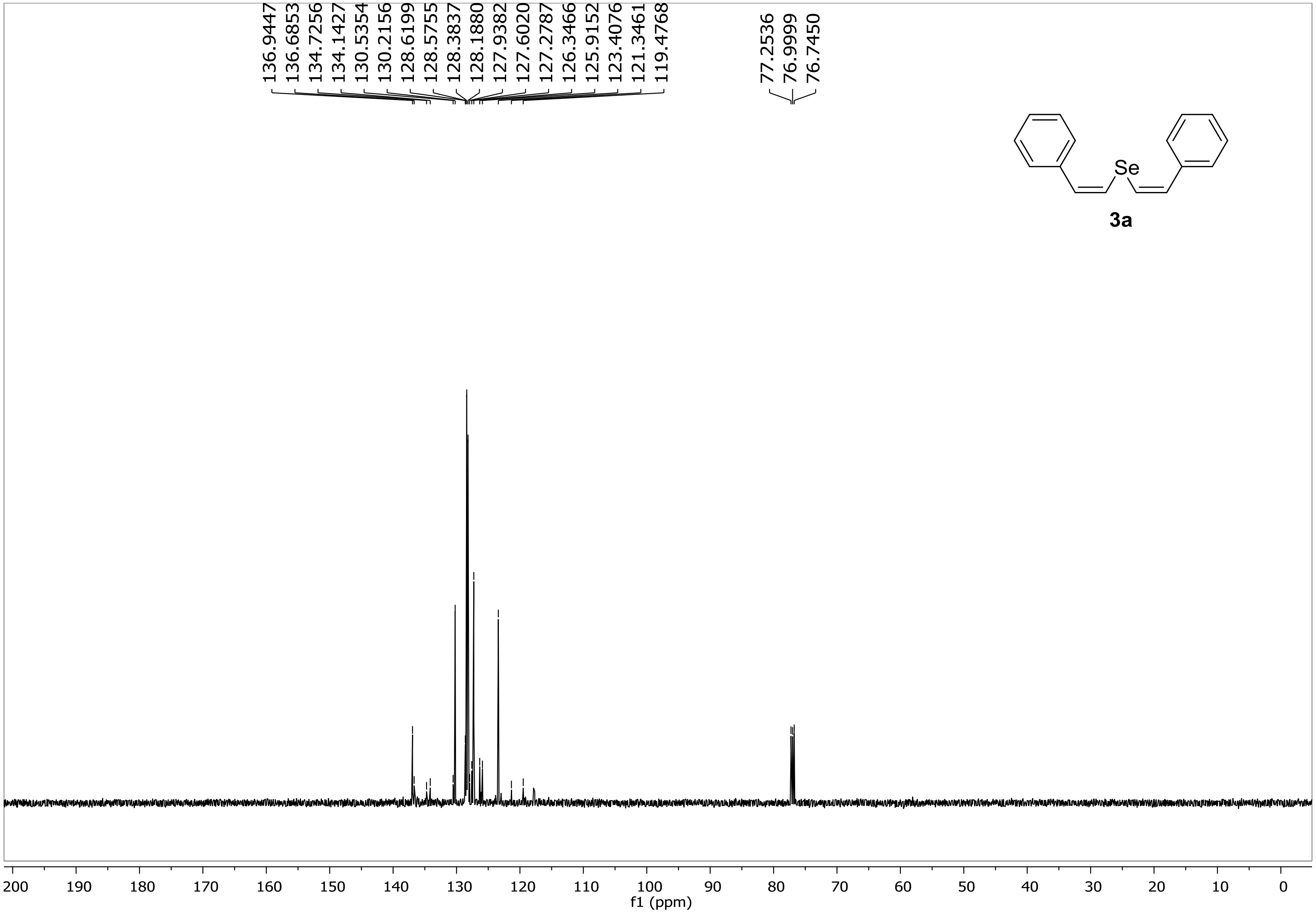

Figure S2. ^{13}C NMR (125 MHz, $CDCl_3$) spectrum of bis-(*Z*,*Z*)-styryl selenide **3a**.

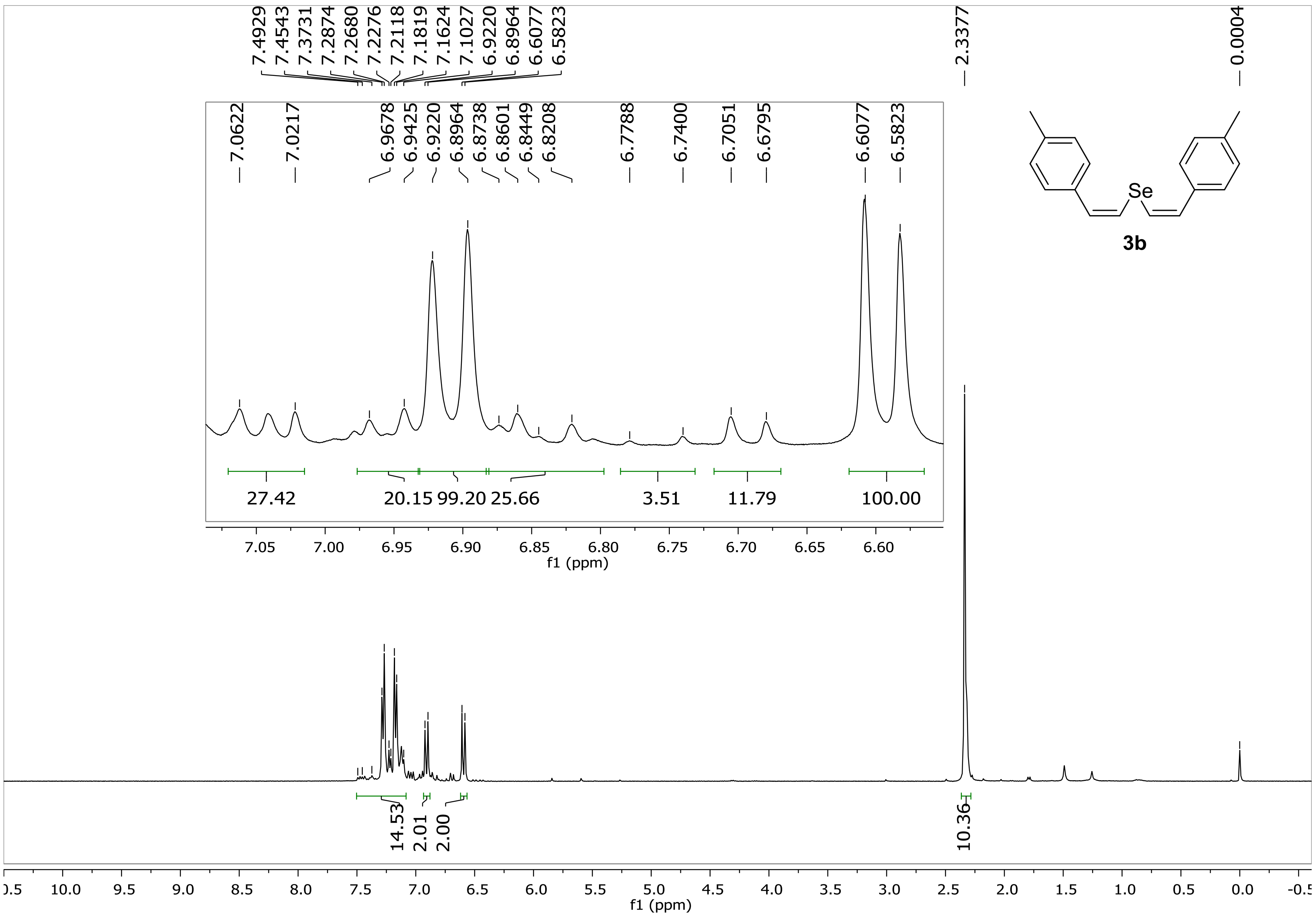

Figure S3. ^{1}H NMR (400 MHz, $CDCl_3$) spectrum of bis-(*Z*,*Z*)-4-methylstyryl selenide **3b**.

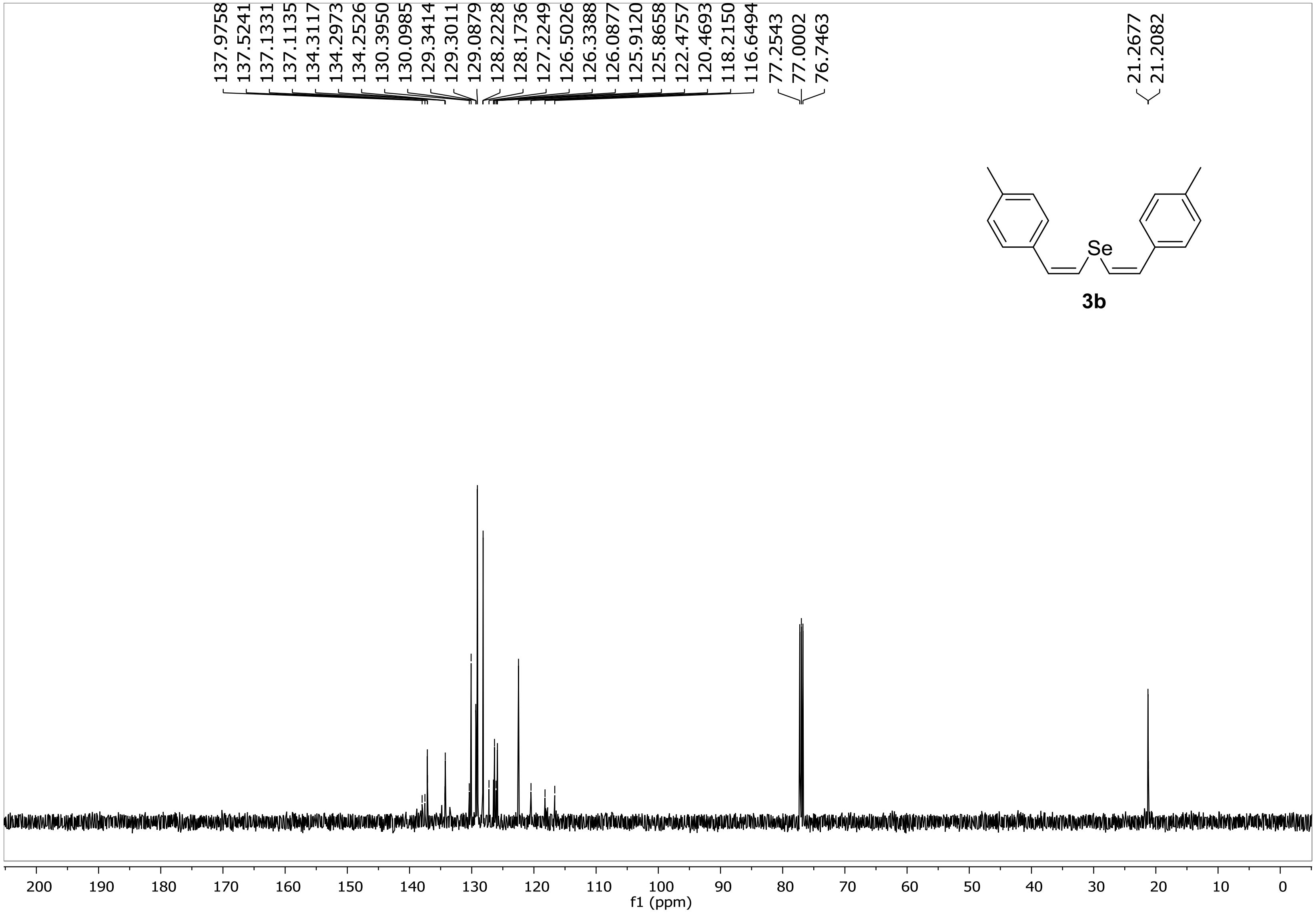

Figure S4. ^{13}C NMR (125 MHz, $CDCl_3$) spectrum of bis-(*Z*,*Z*)-4-methylstyryl selenide **3b**.

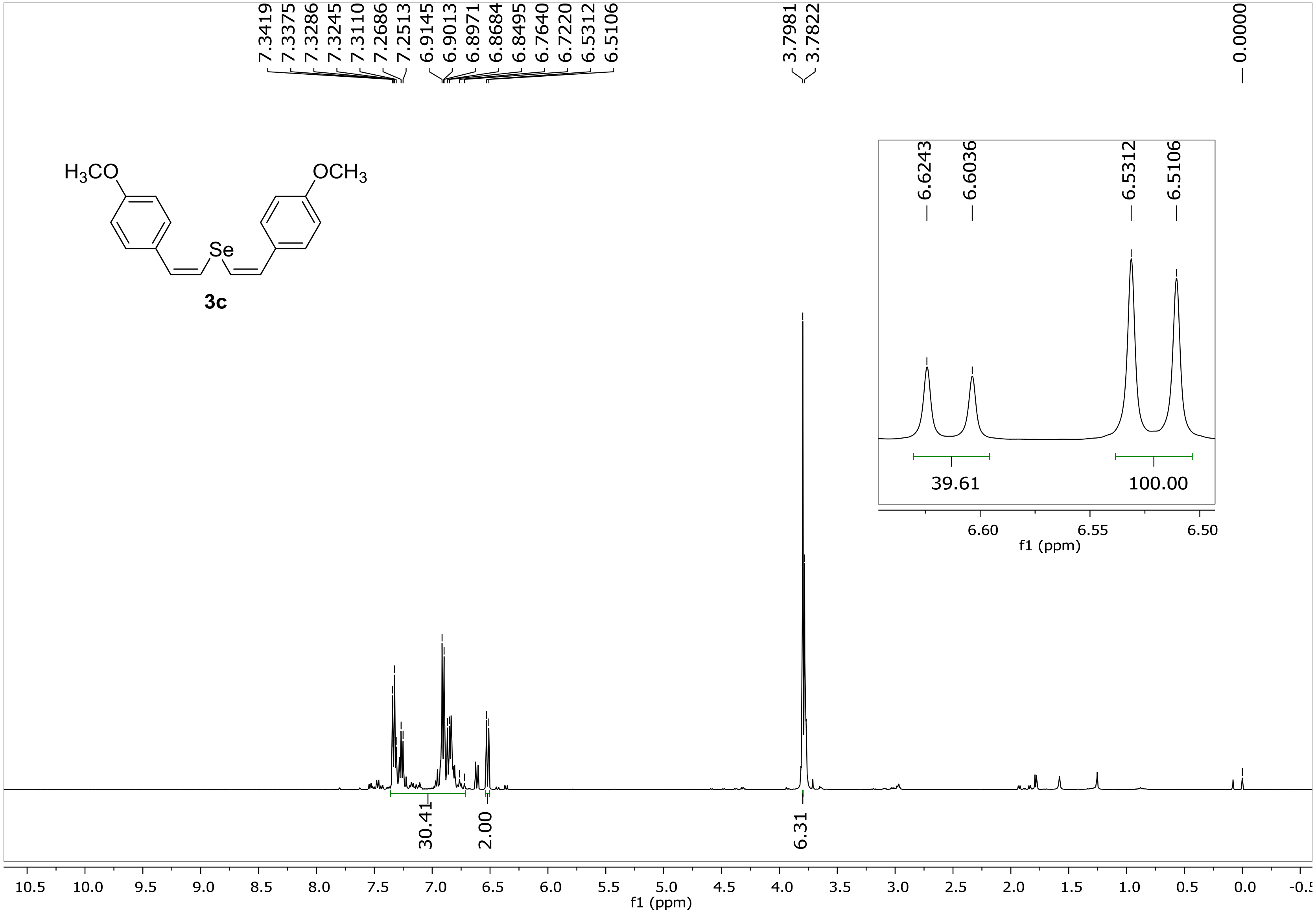

Figure S5. ^{1}H NMR (500 MHz, $CDCl_3$) spectrum of bis-(*Z*,*Z*)-4-methoxystyryl selenide **3c**.

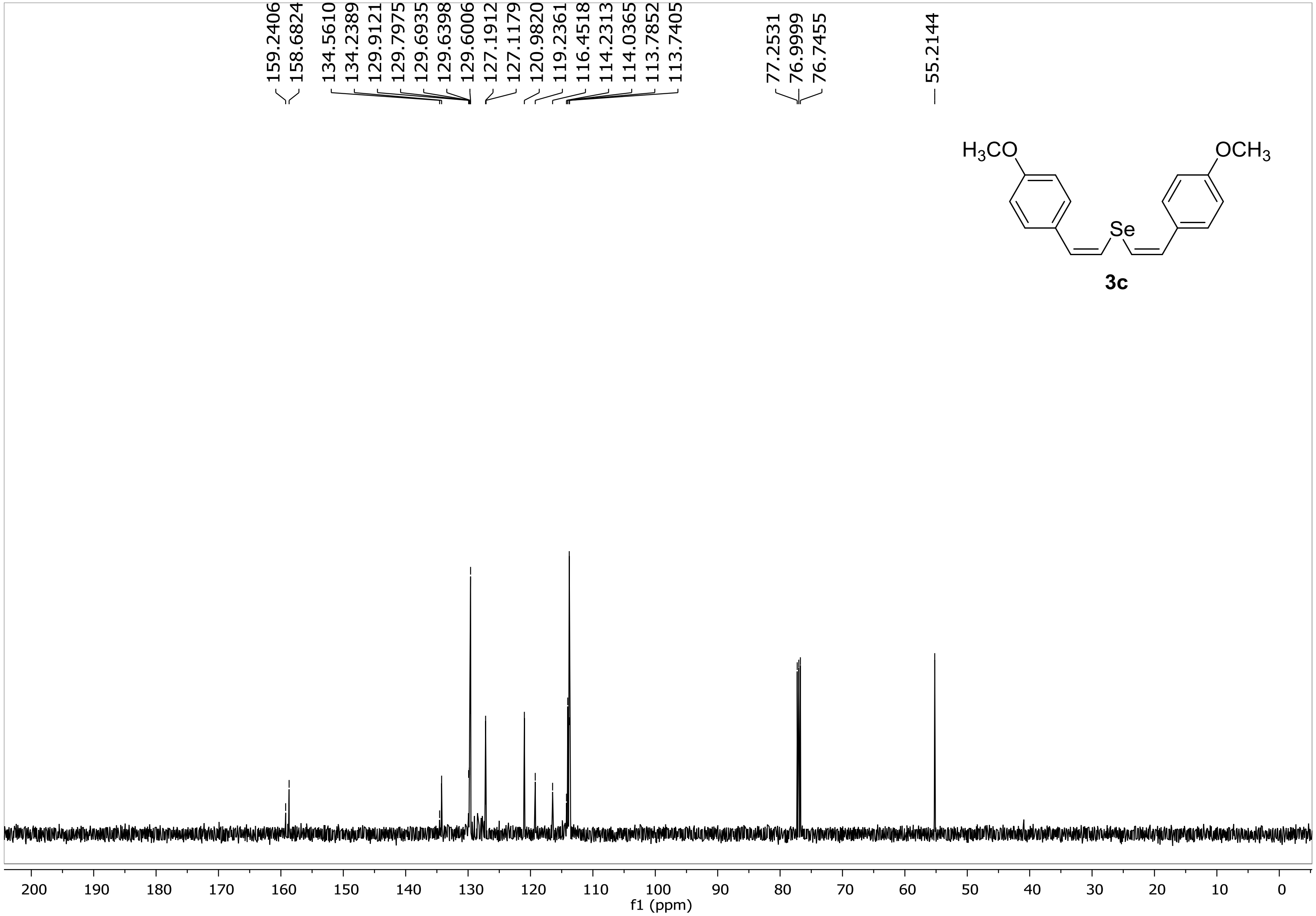

Figure S6. ^{13}C NMR (125 MHz, $CDCl_3$) spectrum of bis-(*Z*,*Z*)-4-methoxystyryl selenide **3c**.

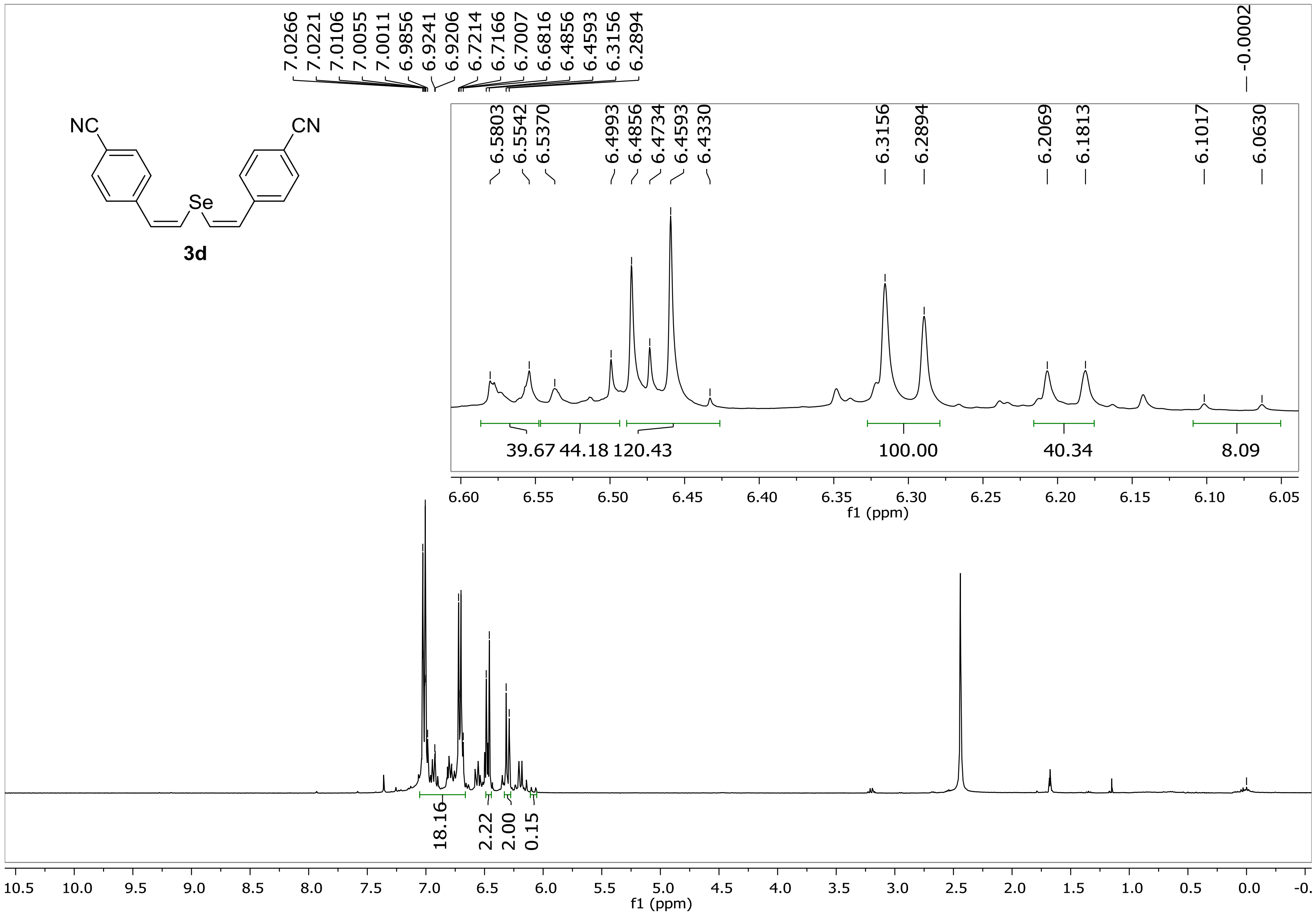
NC
CN
Se
7.0266 7.0221 7.0106 7.0055 7.0011 6.9856 6.9241 6.9206 6.7214 6.7166 6.7007 6.6816 6.4856 6.4593 6.3156 6.2894
-0.0002
6.5803 6.5542 6.5370 6.4993 6.4856 6.4734 6.4593 6.4330 6.3156 6.2894 6.2069 6.1813 6.1017 6.0630
39.67 44.18 120.43 100.00 40.34 8.09
f1 (ppm)
18.16 2.22 2.00 0.15
f1 (ppm)

3d

Figure S7. ^{1}H NMR (400 MHz, DMSO-*d6*) spectrum of bis-(*Z*,*Z*)-4-cyanostyryl selenide **3d**.

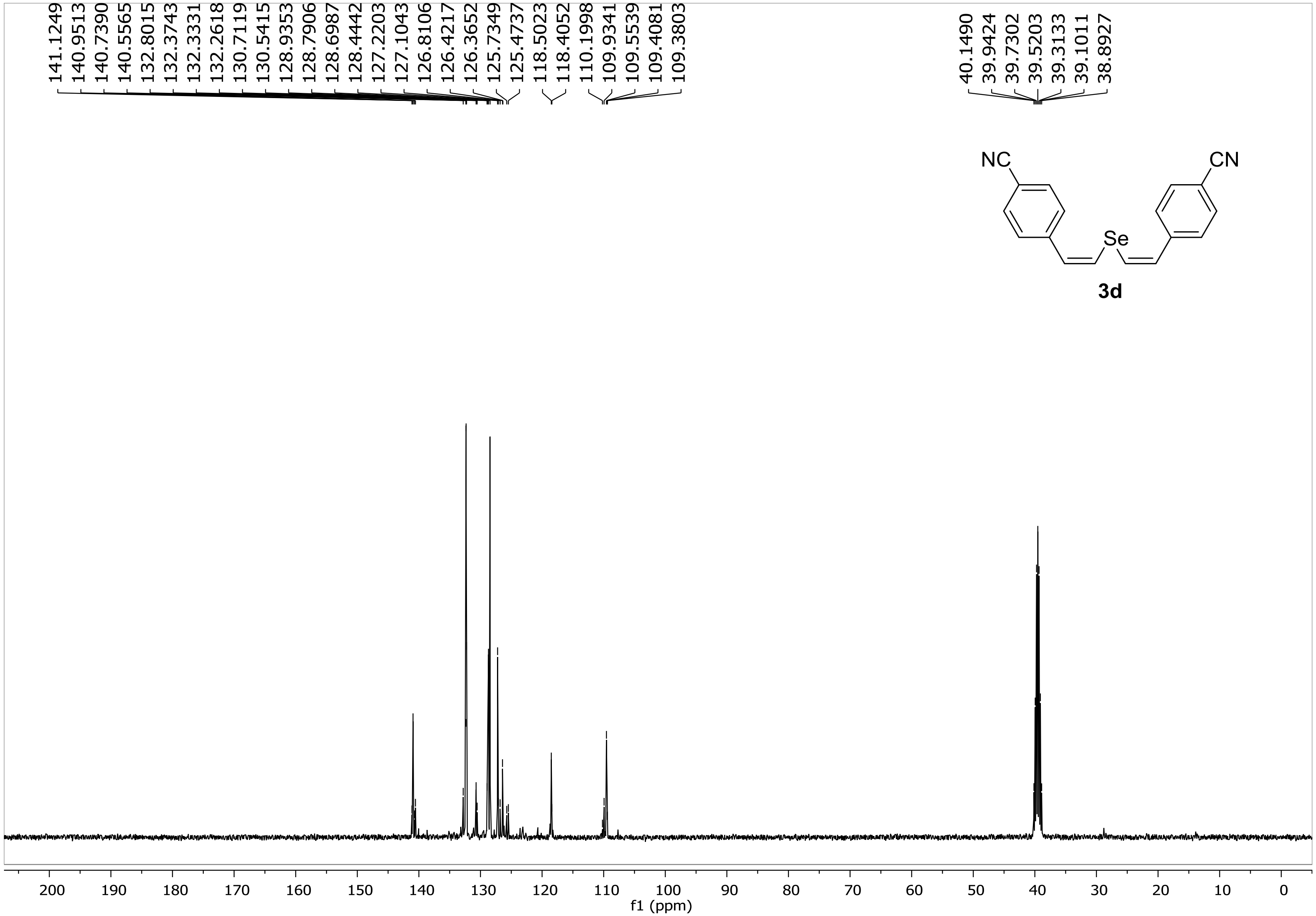

Figure S8. ^{13}C NMR (125 MHz, DMSO-*d6*) spectrum of bis-(*Z*,*Z*)-4-cyanostyryl selenide **3d**.

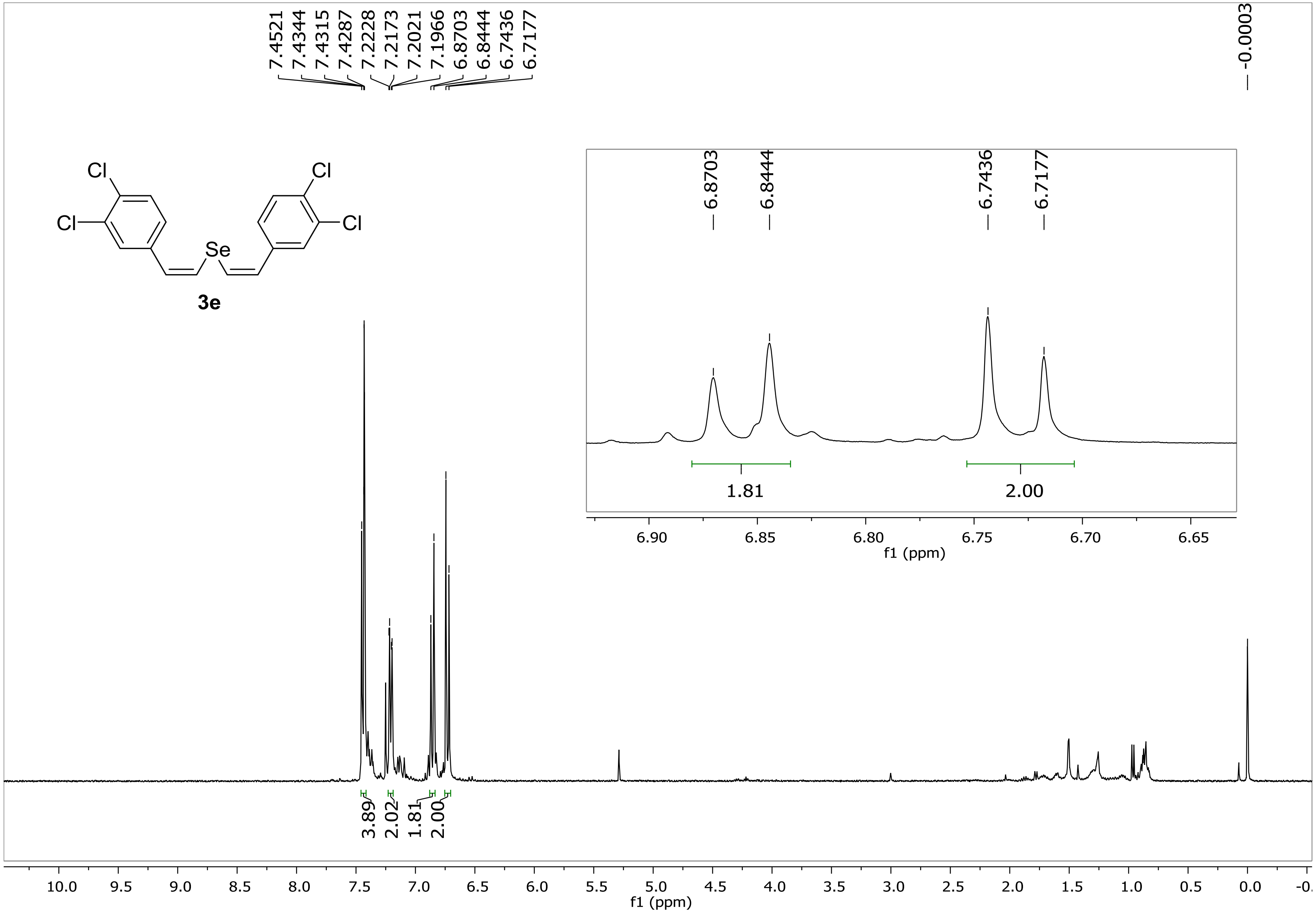

Figure S9. ^{1}H NMR (400 MHz, $CDCl_3$) spectrum of bis-(*Z*,*Z*)-3,4-dichlorostyryl selenide **3e**.

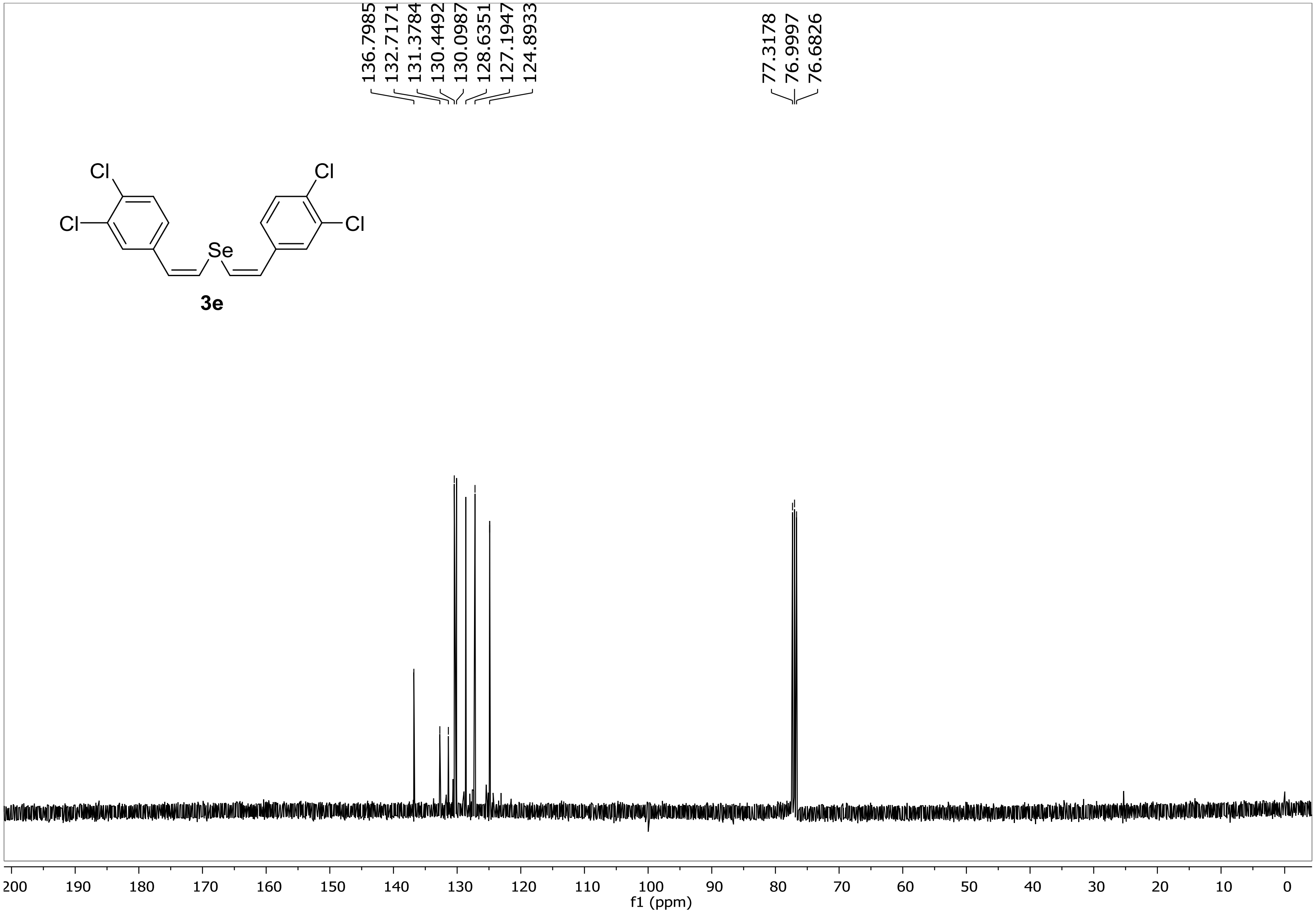

Figure S10. ^{13}C NMR (100 MHz, $CDCl_3$) spectrum of bis-(*Z*,*Z*)-3,4-dichlorostyryl selenide **3e**.

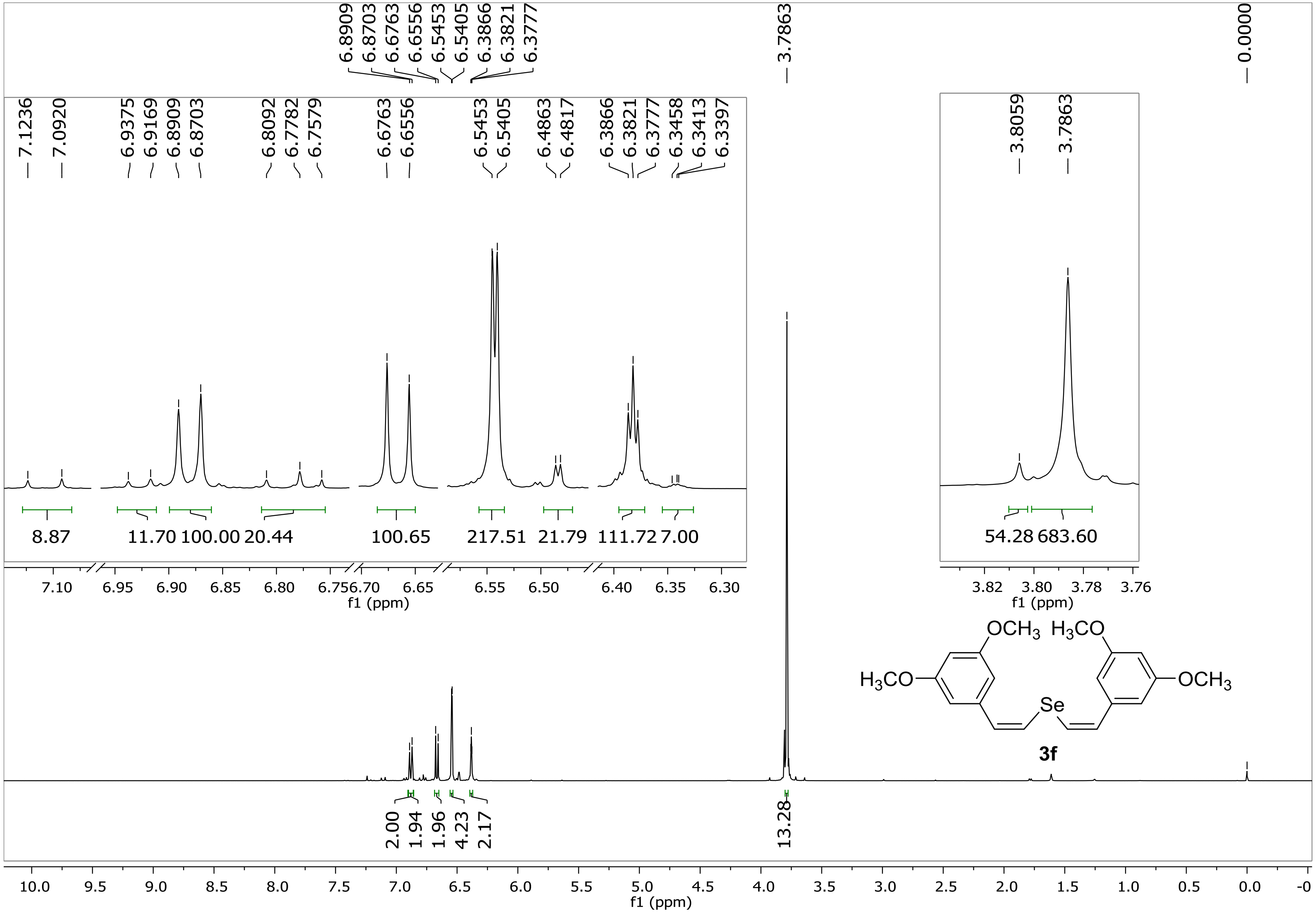

Figure S11. ^{1}H NMR (400 MHz, $CDCl_3$) spectrum of bis-(*Z*,*Z*)-3,5-dimethoxystyryl selenide **3f**.

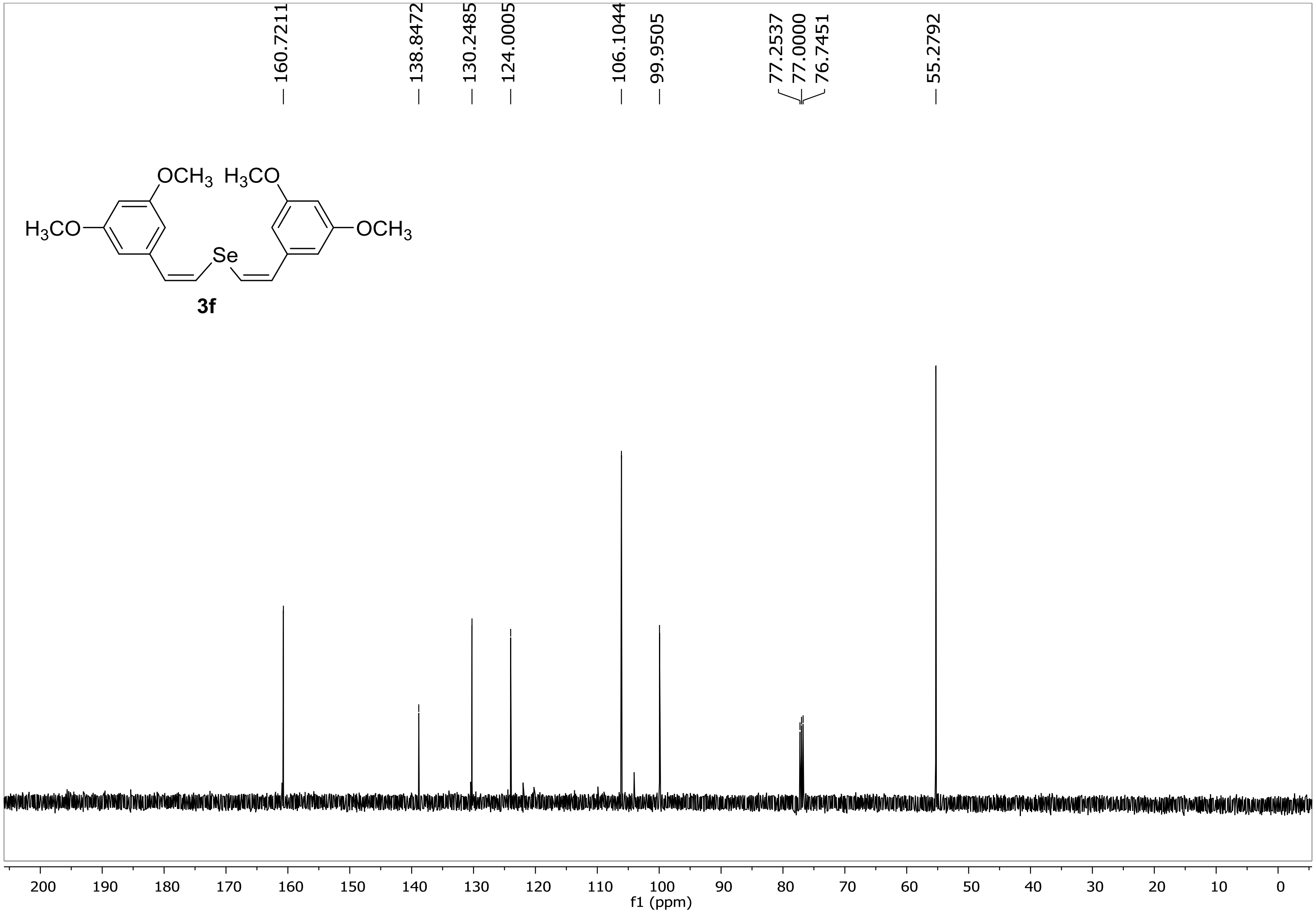

Figure S12. ^{13}C NMR (125 MHz, $CDCl_3$) spectrum of bis-(*Z*,*Z*)-3,5-dimethoxystyryl selenide **3f**.

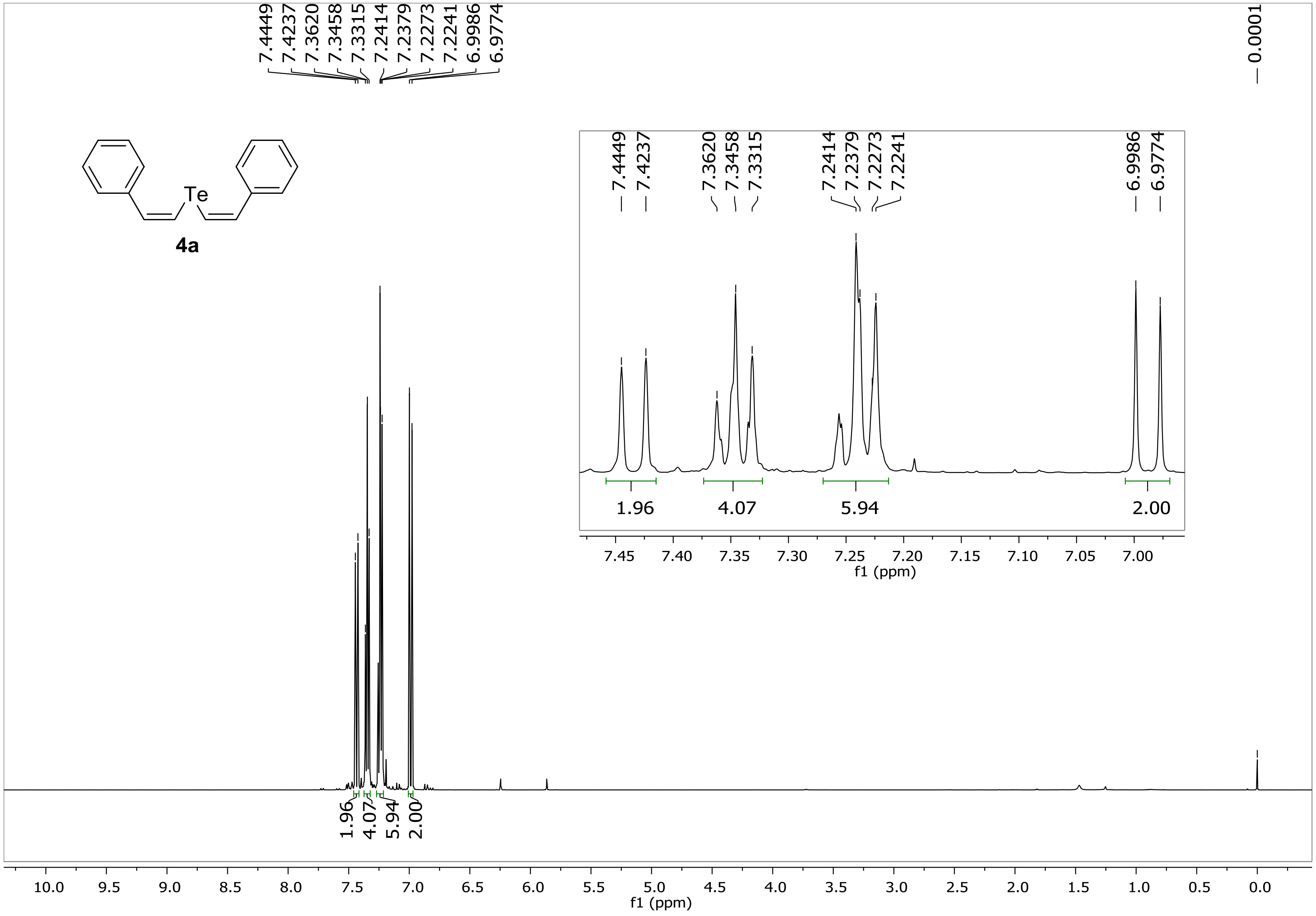

Figure S13. ^{1}H NMR (400 MHz, $CDCl_3$) spectrum of bis-(*Z,Z)*-styryl telluride **4a**.

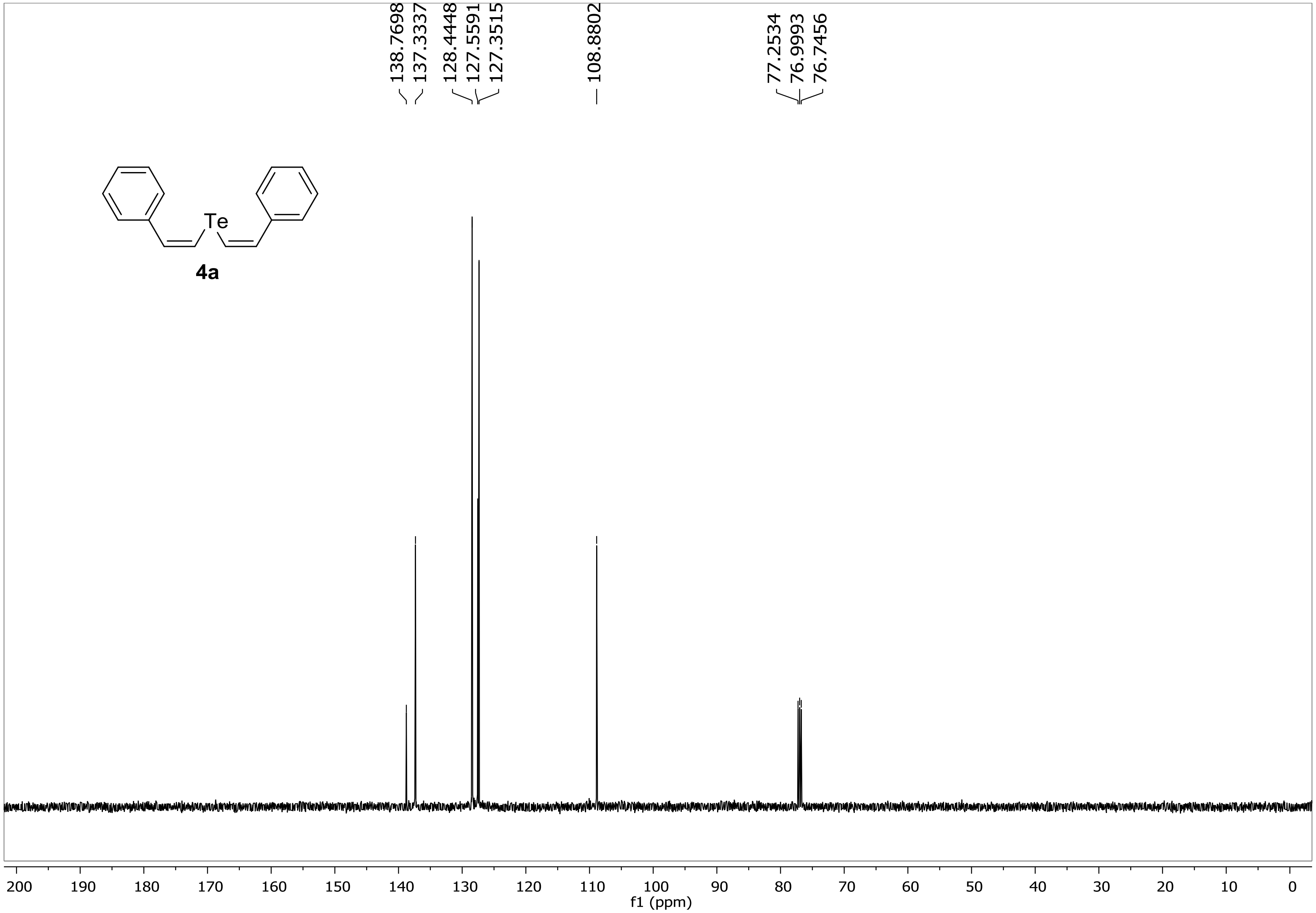

Figure S14. ^{13}C NMR (100 MHz, $CDCl_3$) spectrum of bis-(*Z,Z*)-styryl telluride **4a**.

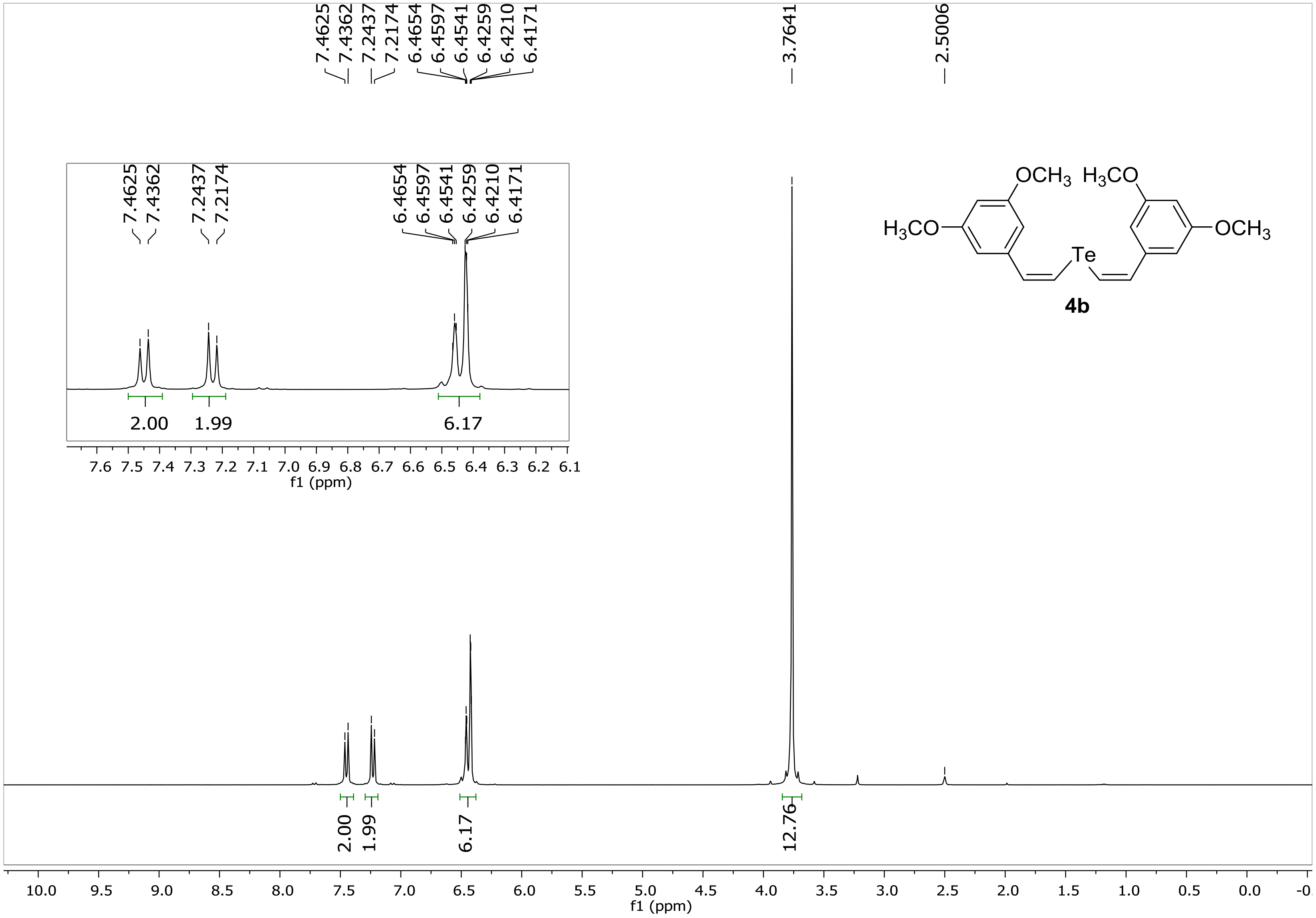

Figure S15. ^{1}H NMR (400 MHz, $CDCl_3$) spectrum of bis-(*Z*,*Z*)-3,5-dimethoxystyryl telluride **4b**.

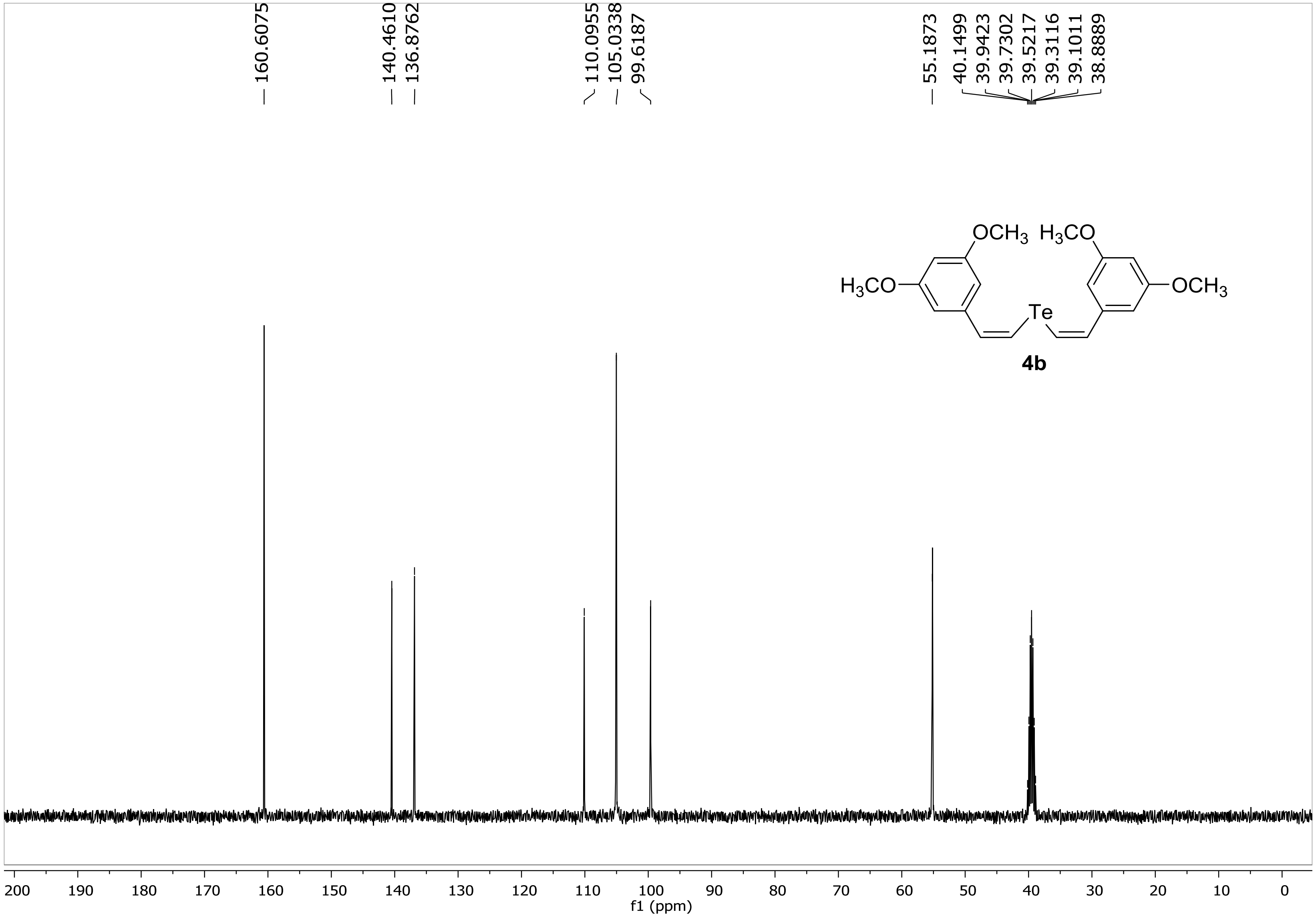

Figure S16. ^{13}C NMR (100 MHz, $CDCl_3$) spectrum of bis-(*Z*,*Z*)-3,5-dimethoxystyryl telluride **4b**.

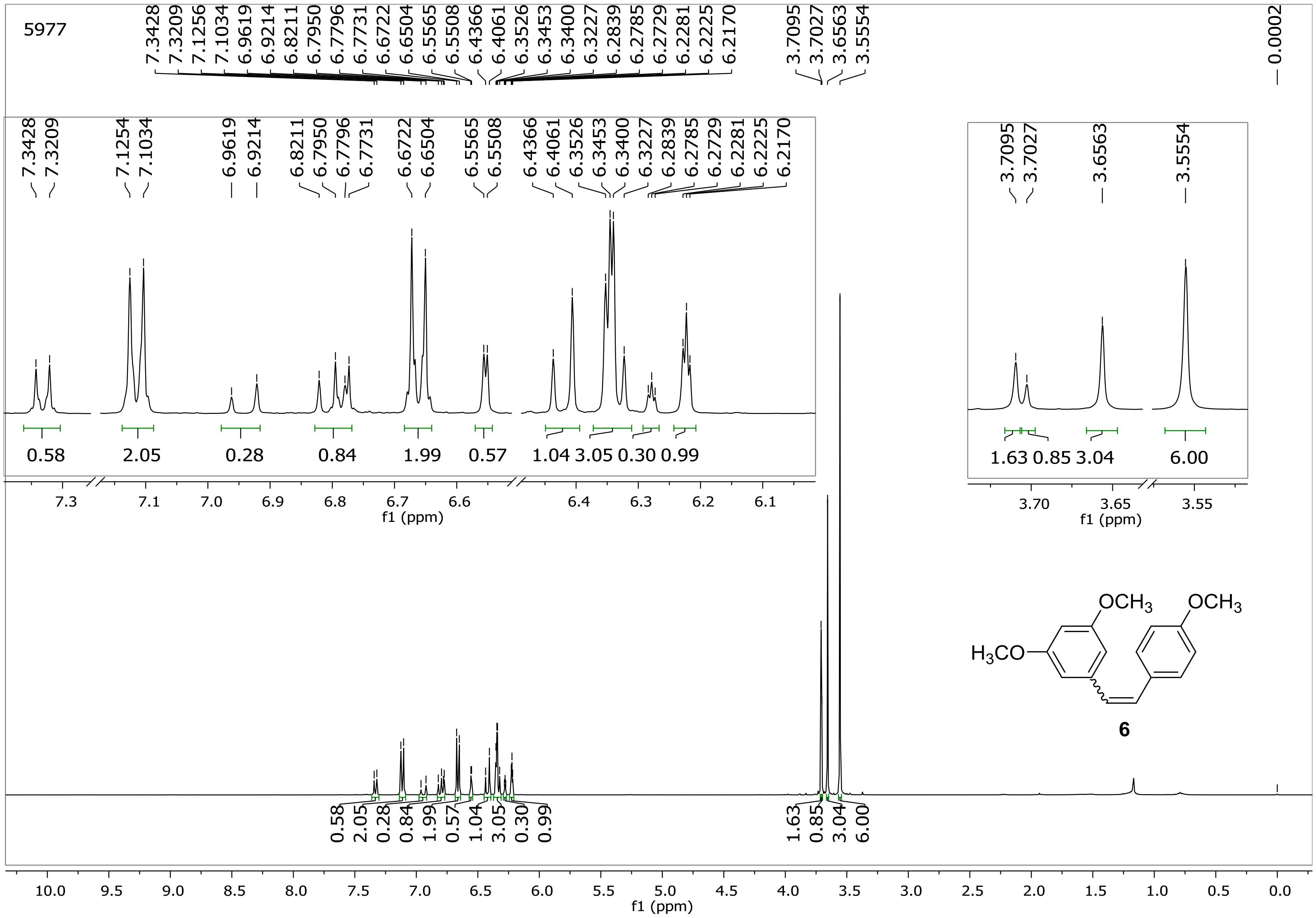
5977
7.3428 7.3209 7.1256 7.1034 6.9619 6.9214 6.8211 6.7950 6.7796 6.7731 6.6722 6.6504 6.5565 6.5508 6.4366 6.4061 6.3526 6.3453 6.3400 6.3227 6.2839 6.2785 6.2729 6.2281 6.2225 6.2170
3.7095 3.7027 3.6563 3.5554
0.0002
0.58 2.05 0.28 0.84 1.99 0.57 1.04 3.05 0.30 0.99
1.63 0.85 3.04 6.00
f1 (ppm)
OCH_3
OCH_3
H_3CO
6

Figure S17. ^{1}H NMR (400 MHz, $CDCl_3$) spectrum of 3,4′,5-trimethoxystilbene **6**.

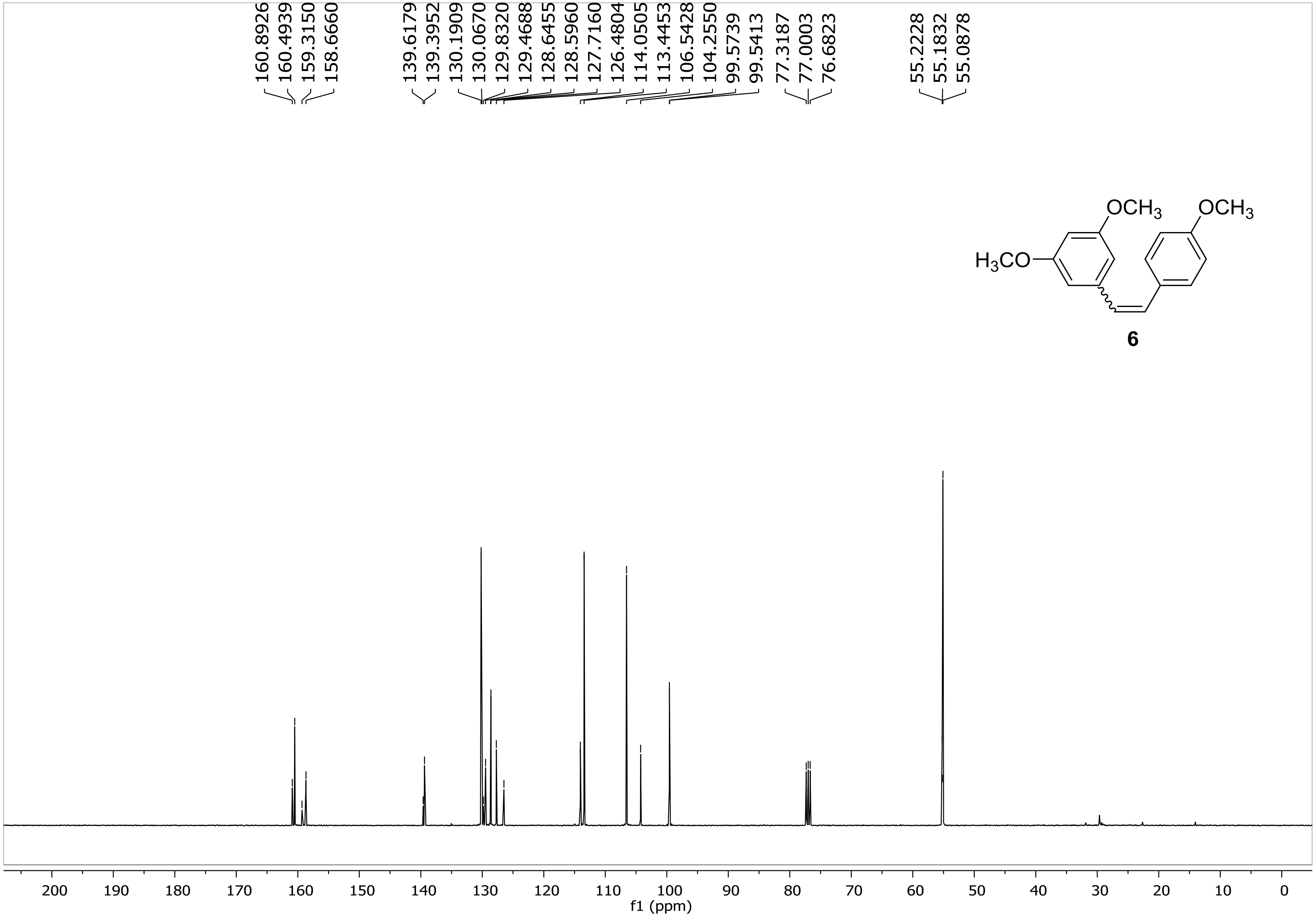

Figure S18. ^{13}C NMR (100 MHz, $CDCl_3$) spectrum of 3,4′,5-trimethoxystilbene **6**.
